# Supplementary material for: Quantum transport evidence of isolated topological nodal-line fermions
Source: Nat Commun. 2022 Nov 23;13:7188. doi: 10.1038/s41467-022-34845-x (PMC9684491; doi:10.1038/s41467-022-34845-x)
Supplement: Supplementary file 1 — Supplementary Information [file 41467_2022_34845_MOESM1_ESM.pdf]

Supplementary Information: Quantum transport evidence of isolated topological nodal-line fermions

Hoil Kim,[1,2] Jong Mok Ok,[1,2] Seyeong Cha,[3] Bo Gyu Jang,[4] Chang Il Kwon,[1,2] Yoshimitsu Kohama,[5] Koichi Kindo,[5] Won Joon Cho,[6] Eun Sang Choi,[7] Youn Jung Jo,[8] Woun Kang,[9] Ji Hoon Shim,[4] Keun Su Kim,[3] and Jun Sung Kim[1,2,*]

[1]*Center for Artificial Low Dimensional Electronic Systems, Institute for Basic Science (IBS), Pohang 37673, Korea*

[2]*Department of Physics, Pohang University of Science and Technology (POSTECH), Pohang 37673, Korea*

[3]*Department of Physics, Yonsei University, Seoul 03722, Korea*

[4]*Department of Chemistry, Pohang University of Science and Technology (POSTECH), Pohang 37673, Korea*

[5]*Institute for Solid State Physics, University of Tokyo, Kashiwa, Chiba 277-8581, Japan*

[6]*Material Research Center, Samsung Advanced Institute of Technology (SAIT), Samsung Electronics Co., Ltd, Suwon-si, Gyeonggi-do 16678, Korea*

[7]*National High Magnetic Field Laboratory, Florida State University, Tallahassee, Florida 32310, USA*

[8]*Department of Physics, Kyungpook National University, Daegu, Korea*

[9]*Department of Physics, Ewha Womans University, Seoul 03760, Republic of Korea*

(Dated: November 3, 2022)

* js.kim@postech.ac.kr

Supplementary Note 1: Crystal growth and characterization

Single crystals of $SrAs_3$ were grown by Bridgman method. A stoichiometric mixture of Sr (99.99%) and As (99.99%) pieces was sealed in an evacuated quartz tube. The quartz tube was then heated up to 550 °C and annealed for 2 hours, followed by slow heating up to 750 °C for 4 days and annealing of 2 days. After characterization using X-ray powder diffraction and energy-dispersive spectroscopy, the precursor powder was sealed in an graphitized quartz ampoule with a conical-shaped tip for controlled nucleation of single crystal growth using the Bridgman method. The evacuated quartz ampoule with precursor powder was suspended and heated up to 800 °C and kept in 1 day for complete melting. Then this ampoule was slowly moved with a speed of 0.5 mm/hour from the hot to the cold zone with a temperature gradient of 5 K/mm. The obtained crystal boules have a cylindrical-rod shape with a diameter, same as the inner one of the ampoule, $\sim$ 12 mm. The X-ray diffraction of the $SrAs_3$ crystal shows sharp $(00l)$ peaks, which confirm high crystallinity of crystals (Supplementary Fig. 1e).

$SrAs_3$ consists of buckled As planes and staked along the c-axis in monoclinic structure (space group $C2/m$) as shwon in Supplementary Figs. 1a and 1b. The As layers form channels in the a–b direction and Sr atoms are inserted into the channels. The mirror plane is defined perpendicular to b axis as indicated by the dashed line in Supplementary Fig. 1b. The scanning transmission electron microscopy (STEM) clearly visualizes the structure of $SrAs_3$ (Supplementary Figs. 1c and 1d). The STEM image normal to the b-axis reveal the buckled network of As atoms, together with the Sr atoms inserted. The magnified view matches well with the crystal structure with lattice constants of $a = 9.604(7)$, $b = 7.651(5)$, $c = 5.869(4)$ Å obtained by X-ray diffraction.

Supplementary Note 2: Electronic structure determination

Figure S2a shows the electronic structures of $SrAs_3$, obtained by the first-principle calculations, based on the generalized gradient approximation (GGA), together with spin-orbit coupling (SOC). A large pocket with a nodal-line is located around the Y point (Supplementary Fig. 2b), and an electron pocket around the T point is observed, consistent with previous band calculations [1]. However, it has been well known that in GGA calcula-

tions, the band-gap of semiconductors is usually underestimated or even absent in some cases. Precise determination of the band gap or band overlap is critical, particularly for nodal-line semimetal (NLSM) candidates with a small single nodal-ring. Furthermore, the presence or absence of the topologically-trivial states at the Fermi level (E_F) drastically affects the transport properties. In previous band calculations, based on GGA or the non-local HeydScuseria-Ernzerhof (HSE06) hybrid functional, the electron bands, located near the T point, are topologically-trivial. In this work, we instead adopted the modified Becke–Johnson exchange potential (mBJ) to overcome the limitation of simple GGA calculation. The mBJ method is known to yield accurate band gap with the computational cost, comparable to the simple GGA calculations [2]. Figure S2c shows the band structures, obtained from mBJ+SOC calculations. The large electron pocket around T point in GGA calculation completely disappears, while the nodal-line states remains around the Y point in mBJ calculations. The conduction and valence band near the Y point are derived from the As1 p and As2 p states (Supplementary Fig. 2d) [1]. These results reveals that the low-energy electronic structures of $SrAs_3$ are extremely sensitive to the details of band calculations, which need to be determined by experiments as discussed below.

In order to identify the electronic structures, we carried out the angle-resolved photoemission spectroscopy (ARPES) in a relatively wide energy range, along k_x and k_z directions. The key features taken along the k_x and k_z directions at high binding energies E_B are well reproduced by the calculations. For examples, the hole band near $k_{x,z} = 0$ with a fast dispersion (Supplementary Fig. 3a), a weakly dispersing bands near $E_\mathrm{B} \sim$ -1.2 eV (Supplementary Figs. 3a and 3d), a highly-dispersive bands near $E_\mathrm{B} \sim$ -2 eV at the zone boundaries (Supplementary Fig. 3d) are consistent with the calculated bands based on both mBJ (Supplementary Figs. 3b and 3e) and GGA (Supplementary Figs. 3c and 3f). However, for the low-energy states, the mBJ calculations captures the ARPES results better than GGA ones, particularly, the absence of the trivial electron bands at the zone boundaries. These observations confirm that the states at the Fermi level are the nodal-line states alone in $SrAs_3$.

Supplementary Note 3: Transport properties and Shubnikov-de Hass oscillations

The transport properties of eleven samples (S1 and S2 in the main text, A1-A9 in the Supplementary Information) with different chemical potentials are characterized. The in-plane resistivity (ρ) of all the samples exhibits a metallic behaviour at high temperatures, while below $T = 50$–70 K, it increases with lowering temperature (Supplementary Fig. 4a). This temperature dependence is due to strong temperature-dependent densities of hole and electron carriers, as discussed in Figs. 1f and 1g in the main text. The field-dependent Hall resistivity $\rho_{xy}(H)$ taken at 2 K indicates that hole carriers are dominant for all samples (Supplementary Fig. 4b). The detailed analysis on $\rho_{xy}(H)$ curves was carried out using the two-band model, as described by $\rho_{xy}(H) = \frac{H}{e}\frac{(n_h\mu_h^2 - n_e\mu_e^2) + (n_h - n_e)\mu_h^2\mu_e^2H^2}{(n_h\mu_h + n_e\mu_e)^2 + (n_h - n_e)^2\mu_h^2\mu_e^2H^2}$, where $n_{e,h}$ and $\mu_{e,h}$ are carrier densities and mobilities for electrons (e) and hole (h), respectively. The fitting results are summarized in Supplementary Table 1. In the samples A1, A2, A3 and S1, only hole carriers contribute to the electronic conduction, producing a linear field-dependent $\rho_{xy}(H)$. On the other hands, the samples A4–A9 and S2 show a slight nonlinearity at low magnetic fields due to a small electron density n_e. Even in these cases, n_e is one or two order of magnitude smaller than the hole density n_h. In these samples, we observed clear Shbnikov-de Hass (SdH) oscillations (Supplementary Figs. 4c and 4d), and the corresponding SdH frequencies are extracted from fast Fourier transform (FFT) analysis (Fig S4e). The SdH frequency increases with increasing the hole carrier density n_h (Supplementary Fig. 4f). These results, together with the relatively good agreement between the size of torus-shaped Fermi surface (Fig. 3a in the main text) and the hole carrier density n_h, support the fact that hole-type nodal-line fermions are responsible for SdH oscillations.

For two representative samples S1 and S2, we obtained SdH oscillations under various magnetic field orientations in three different planes of (k_x,k_z), (k_y,k_z), and (k_x,k_y). The corresponding SdH frequency at different polar (θ) and azimuthal (ϕ) angles is shown in Fig. 3a of the main text. Even in high field experiments using pulse magnetic fields up to ~ 58 T (Supplementary Fig. 5g), additional SdH oscillations were not detected, further confirming that no other FSs except the torus-shaped FS of nodal-line fermions.

To estimate the phase offset of SdH oscillations ϕ_{SdH} using Landau fan diagram, we first compared SdH oscillations in the resistivity ρ_{xx} and the conductivity $\sigma_{xx} = \rho_{xx}/\left(\rho_{xx}^2 + \rho_{xy}^2\right)$ under $H \parallel k_y$, as shown in Supplementary Fig. 6. Because the transverse Hall resistivity $\rho_{xy}(H)$ is much larger than the longitudinal resistivity $\rho_{xx}(H)$ (Supplementary Fig. 6a), SdH oscillations of $\Delta\rho_{xx}(H)$ and $\Delta\sigma_{xx}(H)$ are in-phase (Supplementary Fig. 6b). Thus we

assigned the maxima and minima of the magnetic field-dependent $\Delta\rho(H)$ as integers and half-integers of the Landau index, respectively. Then the phase offset ϕ_{SdH} is extracted from the intercept of the linear fit in the Landau fan diagram (Supplementary Fig. 6c).

Supplementary Note 4: Angle-dependent SdH frequency for torus-shaped Fermi surfaces

The angle dependence of the SdH frequency, particularly as a function of the polar angle (θ) to the k_z axis, is sensitive to the detailed shape of torus-shaped Fermi surface (FS). In an ideal torus-shaped FS of which the poloidal cross-section is of the circular or elliptical shape (Supplementary Fig. 7a), two different SdH frequencies are expected in the whole range of θ. For $\theta = 0°$, two extremal toroidal orbits, inner (β) and outer (δ), correspond to the lower and higher SdH frequencies, respectively. Since the β and δ orbits correspond to the minimum and maximum cross-section, respectively, their SdH frequencies exhibit the opposite angle dependence by tilting the magnetic field off from $\theta = 0°$ (Supplementary Fig. 7c). For $\theta = 90°$, two extremal poloidal orbits, α and γ, which are located on and off the plane of the axis of the torus, respectively. This can be easily seen by plotting the area of the cross-section, generated by an intersecting planes at different k_y, parallel to the torus axis (spiric section), as shown in Supplementary Fig. 7b. Two extrema, one at $k_y = 0$ and the other at $k_y \neq 0$, can be obtained, as indicated by the arrows. The similar angle dependent quantum oscillation frequency has been observed recently on a NLSM candidate, CaAgAs [3, 4].

Unlike the ideal case, the resulting torus-shaped FS of $SrAs_3$ has the poloidal cross-section of the crescent shape, not the circle shape (Supplementary Fig. 7d). There are three key differences in the angle (θ) dependent SdH frequency, as compared to the case of the ideal torus-shaped FS. Firstly, at $\theta = 0°$, both β and δ orbits correspond to the maximum cross-section. Therefore, near $\theta = 0°$, the SdH frequencies of the β and δ orbits exhibit the same angle dependence (Supplementary Fig. 7f). Secondly, for $\theta = 90°$, only one extremal cross-section can be found on the plane of the axis of the torus, which corresponds to the α orbit (Supplementary Fig. 7e). There is no extremal orbits away from the center of the torus, and thus the SdH oscillations corresponding to the γ orbit, found in the ideal torus case, are missing. Both features are well reproduced in experiments on $SrAs_3$ (Fig. 3a in

the main text).

For the torus FS with crescent-shaped cross-section, additional inner extremal orbits (β') also can be formed above and below the nodal line plane (Supplementary Fig. 8a). According to our model Hamiltonian, additional SdH oscillation frequency $\beta' \sim 18$ T is expected in the $SrAs_3$ crystal as shown in the Supplementary Fig. 8b. This β' oscillations, however, were not detected in experiments. The key parameters determining the amplitude of SdH oscillation are curvature factor $C = |\partial^2 A/\partial k_{||}^2|^{-1/2}$ and cyclotron effective mass m^*, where A is enclosed k-space area and $k_{||}$ is k component parallel to the magnetic field direction [4]. In our model Hamiltonian of $SrAs_3$, the β' orbit has smaller curvature factor $C \sim 0.053$ and larger cyclotron mass $m^* \sim 0.619 m_e$ than those of the β orbit with $C \sim 0.103$ and $m^* \sim 0.246 m_e$. Assuming that additional β' orbit has dingle temperature $T_D \sim 12$ K and g-factor ~ 3, similar to those of the β orbit obtained from SdH oscillations, we can estimate the expected oscillation amplitude of the β' orbit using the Lifshitz–Kosevich (LK) formula [5, 6] at $T = 1.8$ K and $H = 15$ T. Its amplitude is ~100 times smaller than that of the β orbit, which is well below our detection limit. Therefore, we focus on the β orbit in the main text.

Supplementary Note 5: Spin-splitting effect on SdH oscillations

In order to estimate the phase ϕ_0 in Eq. (1) of the main text, which is related to the Berry phase ϕ_B, it is important to determine the additional phase ϕ_s by the spin-splitting effect in SdH oscillations. As described in Eq. (1) the spin-splitting of the Landau levels (LLs) by the Zeeman effect introduces the phase shift by $\pm\phi_s = \pm g m^*/2m_e$, where g is the g-factor, m^* is the effective mass, and m_e is the free electron mass. When the spin-splitting phase ϕ_s becomes closer to $\sim 1/2$, SdH oscillations are phase-shifted by π, which can be confused with π Berry phase shift in ϕ_0. In $SrAs_3$, however, SdH oscillations at high magnetic fields, near the quantum limit, exhibit additional features in $\rho(H)$ due to this spin splitting of LLs. As clearly visible in the second-derivative of $\rho(H)$, $-d^2\rho(H)/dH^2$ in Figs. 3h and 3i in the main text, the spin-splitting peaks, denoted + and −, are well resolved in SdH oscillations, and their difference allow us to directly estimate ϕ_s for each SdH oscillations.

Before presenting the results from the detailed analysis, it is important to justify the validity of Eq. (1) for Dirac bands. It has been known that for parabolic bands, quantum

oscillations exhibit the constant Zeeman splitting when plotted as a function of $1/H$, because energy differences by Zeeman splitting and Landau level splitting depend linearly on external magnetic field H [7]. This is also the case for Dirac bands when $2\hbar e v_0^2 H \gg (g\mu_B H/2)^2$, where μ_B is Bohr magneton, $\hbar$ is the reduced Plank constant and v_0 is the band velocity [7]. In this work, even at the maximum magnetic field of 31.6 T and for the largest g-factor ~ 19.1, $2\hbar e v_0^2 H$ is an order of magnitude larger than $(g\mu_B H/2)^2$, guaranteeing the validity of Eq. (1). To further clarify this issue, we plot the Landau fan diagram, including the spin-split Landau levels (Supplementary Fig. 9). In Shubnikov-de Hass oscillations, two Zeeman-split peaks in the second derivative of the oscillating magnetoresistivity, $d^2\rho/dH^2$, as a function of $1/H$ are separate by a spacing of ϕ_s/F. In $SrAs_3$, when the Zeeman splitting is smaller than the spin-degenerate Landau level spacing ($1/F$) *i.e.* $\phi_s \sim 0$–0.3, disorder-induced broadening makes two peaks in the oscillating magnetoresistivity merge into one peak, preventing experimental determination of the Zeeman splitting. For $\phi_s \sim 0.35$-0.5, however, the Zeeman splitting spacing become large enough to be detected (Supplementary Figs. 9b, 9d and 9f), and we assigned the middle point of the two Zeeman-split peaks with the integer Landau index. We note that the spin-splitting appears to be constant in the Landau fan diagram as indicated by a vertical bars in Supplementary Figs. 9d–9g, consistent with the discussion above. The ϕ_s is then extracted from the average spin-splitting spacing multiplied by the SdH frequency F. In addition, the linear fitting in the Landau fan diagram shows excellent agreement with the corresponding R-square value > 0.99. This is also the case for larger Zeeman splitting with $\phi_s > 0.5$ (Supplementary Figs. 9c, 9e and 9g), in which the spin-split Landau levels with different orbital Landau indices, such as 3+ and 4−, become close and eventually produce one peak in $d^2\rho/dH^2$ curve (Supplementary Fig. 9e). In this case, we assigned the deep position with the integer index, which again follows a linear dependence in the Landau fan diagram with R-square > 0.99.

Having justified the validity of Eq. (1) for the Dirac bands, we present magnetic field-dependent SdH oscillations with spin splittings at different polar (θ) and azimuthal (ϕ) angles for the α and β orbits in Supplementary Fig. 10. We observed systematic variation of the spin-splitting signatures as a function of angles, indicated by the shaded lines in Supplementary Fig. 10. The spin-splitting features in the SdH oscillations are more clearly seen in S2 than in S1, but their angle dependence are consistent with each other (Supplementary Figs. 10a–10d). Using the obtained ϕ_s and the effective mass m^* (Supplementary Table

2), from temperature dependent SdH oscillations shown in Figs. 2d-2f of the main text, we estimated the g-factors at different magnetic field orientations for the sample S1 (S2): $g \approx 2$ (2) for $H \parallel k_x$, 18.7 (19.1) for $H \parallel k_y$ and 2.61 (3.26) for $H \parallel k_z$. This variation of the g-factor, depending on field orientations, is comparable with other topological semimetals [4, 8]. For the γ orbit with a high SdH frequency of $F > 125$ T, it is hard to identify the spin splitting features in the SdH oscillations. Assuming the same g-factor $\sim 2-3$ with that obtained for the β orbit, we can estimate $\phi_s = gm^*/2m_e$ using the obtained effective mass of γ orbit at $H \parallel k_z$ (Supplementary Table 2). The resulting $\phi_s \sim 0.1$ for the γ orbit is small enough to neglect the spin-splitting effect on the phase offset in SdH oscillations. The phase ϕ_0 is then the same as ϕ_{SdH} from experiments.

Supplementary Note 6: Weak antilocalization analysis.

In addition to weak antilocalization (WAL), two other contributions determine the magnetic field-dependent behaviour of the conductivity in topological semimetals, the orbital magnetoresistance (MR) and chiral anomaly effects. It has been well established that in the transverse configuration $H \perp J$, the orbital MR effect is significant in the absence of the chiral anomaly effect, whereas in the longitudinal configuration $H \parallel J$, the chiral anomaly effect becomes dominant over the much suppressed orbital MR effect. Therefore, in any configurations of the relative directions of the applied magnetic field H and current J, we need to extract the WAL contribution from the others' contributions. At zero magnetic field, the electrical conductivity σ of topological semimetals is described by $\sigma = \sigma_0 + \Delta\sigma_{\mathrm{WAL}}$, where $\Delta\sigma_{\mathrm{WAL}}$ is the excess conductivity due to quantum interference effect, mainly WAL, and σ_0 is the semi-classical conductivity. Under external magnetic fields, $\sigma_0(H)$ exhibits the characteristic magnetic field dependence due to the orbital MR contribution, $\sigma_0^{\mathrm{orb.}}(H) = \frac{1}{\rho_0 + AH^2}$, or the chiral anomaly effect $\sigma_0^{\mathrm{chiral}}(H) = C_w H^2$, where $\rho_0 = 1/\sigma_0(0)$, and A (C_w) is a the coefficient quantifying the strength of the orbital MR (chiral anomlay) effect [9]. In our cases, ρ_0 is three orders of magnitude larger than AH^2 ($\rho_0 \gg AH^2$) within the measured magnetic field range, and thus the orbital MR term can be approximated to be $\sigma_0^{\mathrm{orb.}}(H) = \sigma_0 - AH^2$.

Then the resulting magnetic field-dependent conductivity $\sigma(H)$ is described as

$$\sigma(H) = \sigma_0^{\text{orb.}}(H) + \sigma_0^{\text{chiral}}(H) + \Delta\sigma_{\text{WAL}}(H) = \sigma_0 - AH^2 + C_w H^2 + \Delta\sigma_{\text{WAL}}(H), \quad \text{(Supplementary 1)}$$

which clearly shows that both the orbital MR and chiral anomaly contributions follow H^2 dependence. This contrasts to $\sim \sqrt{H}$ or $\sim \ln H$ dependence of $\Delta\sigma_{\text{WAL}}(H)$ in the WAL for Weyl/Dirac semimetals [10] or nodal-line semimetals [11], respectively. Thus at low magnetic fields, $\Delta\sigma_{\text{WAL}}(H)$ dominates over the H^2-dependent orbital MR and chiral anomaly effects. As presented in Supplementary Fig. 11 for various topological semimetals, a sharp peak in $\Delta\sigma(H)$ at low magnetic fields can be easily distinguished from the slowly-varying contributions from the orbital MR or the chiral anomaly effect [12–15]. Based on Eq. (Supplementary 1), we fit the background H^2 dependent conductivity $\sigma_0(H) = \sigma_0 - AH^2 + C_w H^2$ from the high field data in the transverse or longitudinal configurations, assuming $C_w = 0$ or $A = 0$. Then the remaining contribution in the conductivity corresponds to $\Delta\sigma_{\text{WAL}}(H)$ as shown in Supplementary Fig. 11. Using the obtained $\sigma_0(H)$ and $\Delta\sigma_{\text{WAL}}(H)$, we estimate their zero magnetic field values, σ_0 and $\Delta\sigma_{\text{WAL}}$, which are compared in Supplementary Fig. 11i.

As shown in Supplementary Fig. 11i, we found a decreasing trend of $\Delta\sigma_{\text{WAL}}$ with lowering σ_0. This behaviour is opposite to the simple expectation on the WAL of three-dimensional (3D) disordered metals [16]. It has been well known that the excess conductivity $\Delta\sigma_{\text{WAL}}$ is determined by quantum conductance e^2/h, a degeneracy factor, the elastic mean free path (l_e), and the phase coherence length (l_ϕ). At low temperatures, l_ϕ is much larger than l_e ($l_\phi \gg l_e$), and $\Delta\sigma_{\text{WAL}}$ is inversely proportional to the mean free path l_e. Thus $\Delta\sigma_{\text{WAL}}$ is expected to be larger in the highly resistive samples with a smaller σ_0 [16]. This is opposite to the observed trend between $\Delta\sigma_{\text{WAL}}$ and σ_0 (Supplementary Fig. 11i). As discussed in the main text, in topological semimetals, *e.g.* Weyl semimetals, the size of $\Delta\sigma_{\text{WAL}}$ is very sensitive to detailed balance between intra- and inter-valley scattering processes [10]. This is because the small-angle intravalley scattering favors the WAL due to associated Berry phase, whereas the large-angle intervalley scattering induces the competing weak localization (WL). Similarly, in nodal-line semimetals, quantum interference by small-angle scatterings on the poloidal plane induces the WAL, which is suppressed by the large-angle scatterings along the toroidal direction of the torus-shaped FS [11]. Roughly speaking, the large-angle

scattering is effective to reduce both the excess conductivity $\Delta\sigma_{\rm WAL}$ and the semi-classical conductivity σ_0, resulting in smaller $\Delta\sigma_{\rm WAL}$ with smaller σ_0, as observed experimentally (Supplementary Fig. 11i).

Supplementary Note 7: Dimensionality of weak antilocalization in $SrAs_3$

The magnetoconductivity due to the WAL exhibits the distinct behaviors, depending on the dimensionality. For the tubular FS of nodal-line semimetal, the WAL effect should follow the two-dimensional (2D) behavior when small-angle scatterings are dominant in the poloidal plane [11]. Such a prediction can be tested by examining the magnetoconductivity $\Delta\sigma(H)$ and also the temperature-dependent phase coherence length l_ϕ. For the 2D WAL, the magnetoconductivity $\sigma(H)$ is described by the Hikami-Larkin-Nagaoka model (HLN model), $\Delta\sigma(H) = -\alpha\frac{e^2}{2\pi^2\hbar}\left[\psi(\frac{1}{2}+\frac{B_\phi}{H}) - \ln(\frac{B_\phi}{H})\right]$, where ψ is digamma function and $B_\phi = \hbar^2/(4el_\phi^2)$ is characteristic field associated with phase coherence length l_ϕ [17]. In contrast, the 3D WAL of topological semimetals can be described by the following equation, $\sigma(H) \sim \frac{2e^2}{h}\int_0^{1/l}\frac{dx}{(2\pi)^2}\left[\psi(\frac{l_B^2}{l^2}+l_B^2x^2+\frac{1}{2}) - \psi(\frac{l_B^2}{l_\phi^2}+l_B^2x^2+\frac{1}{2})\right]$ with l the mean free path and $l_B = \sqrt{\hbar/4eH}$ the magnetic length [10]. The 2D WAL model roughly follows $-\ln H$ dependence, whereas the 3D WAL model shows almost $-\sqrt{H}$ dependence. As shown in Supplementary Figs. 12a and 12b, the stiff drops of $\Delta\sigma(H)$ at low magnetic fields is well reproduced by the 2D WAL model ($\sim -\ln H$) rather than the 3D WAL model ($\sim -\sqrt{H}$) for all samples of $SrAs_3$.

The temperature dependence of phase coherence length l_ϕ further supports the same conclusion. For the 2D WAL model, the phase coherence length follows $l_\phi \propto T^{-p/2}$ dependence with the exponents $p = 1$ or $p = 2$ due to electron-electron or electron-phonon interactions, respectively. This contrasts to the 3D WAL behaviors described by different exponents, $p = 3/2$ or $p = 3$ for electron-electron or electron-phonon interactions, respectively. As shown in Supplementary Figs. 12c and 12d, the temperature-dependent $l_\phi \propto T^{-1}$ is observed at high temperatures corresponding to the exponent of $p = 2$ for the 2D electron-phonon interactions. In more details, temperature dependent l_ϕ data in 2D diffusive system can be fit to the following expression, $1/l_\phi^2 = 1/l_{\phi0}^2 + A_{ee}T + A_{ep}T^2$ where $l_{\phi0}$ is zero-temperature dephasing length, A_{ee} and A_{ep} are the coefficients for electron-electron and electron-phonon scatterings, respectively [18]. The best fit, shown in the Supplementary Fig. 12d, reproduces nicely the

experimental data, yielding $l_{\phi 0}$=83(1) nm, $A_{ee} \approx 0$ and $A_{ep} = 7.0(6)\times10^{-8}\text{nm}^{-2}\text{K}^{-2}$, which shows clear 2D WAL with dominant electron-phonon interaction. These results strongly suggest that the observed magnetoconductivity of the bulk $SrAs_3$ crystals agrees well with the theoretically-predicted 2D WAL behaviors of nodal-line semimetals [11].

Supplementary Note 8: Comparison with other nodal-line semimetal candidates.

In Supplementary Table 3, we present the characteristics of various NLSM candidates, including $PbTaSe_2$ [19], ZrSi*Ch* (*Ch* = S, Se, Te) [20–25], HfSiS [26], CaAg*Pn* (*Pn* = P and As) [3, 4, 15, 27], and $SrAs_3$. One distinct character of $SrAs_3$ from the most of NLSM candidates is a single nodal-loop structure. For examples, $PbTaSe_2$ possesses several nodal rings near the Fermi level, together with topologically trivial states [19], which results quantum oscillations with a high frequency $F \sim 1000$ T and the large carrier density. ZrSi*Ch* has a cage-like structure of multiple nodal loops [28, 29] forming a complex nodal-line structures, which are revealed by multiple frequencies of magnetic quantum oscillations, indicating thin and thick tubular FSs coexisting [20–25]. CaAg*Pn* is a rare example possessing a single nodal-loop, similar to $SrAs_3$, as confirmed by recent studies on quantum oscillations [3, 4]. Even for CaAg*Pn*, the polidal cross-section of the torus-shaped FS is estimated to be much larger than that of $SrAs_3$. The corresponding quantum oscillation frequency for the poloidal orbit is $\sim$ 50 - 95 T for CaAgAs [3, 4] much larger than $\sim$ 7 - 13 T for $SrAs_3$. Therefore, among the NLSM candidates so far known, $SrAs_3$ has the thinnest tubular FS.

---

[1] Xu, Q., Yu, R., Fang, Z., Dai, X. & Weng, H. Topological nodal line semimetals in the CaP_3 family of materials. *Phys. Rev. B* **95**, 045136 (2017).

[2] Tran, F. & Blaha, P. Accurate Band Gaps of Semiconductors and Insulators with a Semilocal Exchange-Correlation Potential. *Phys. Rev. Lett.* **102**, 226401 (2009).

[3] Hirose, H. T. *et al.* Real spin and pseudospin topologies in the noncentrosymmetric topological nodal-line semimetal CaAgAs. *Phys. Rev. B* **101**, 245104 (2020).

[4] Kwan, Y. H. *et al.* Quantum oscillations probe the Fermi surface topology of the nodal-line

semimetal CaAgAs. *Phys. Rev. Research* **2**, 012055 (2020).

[5] Shoenberg, D. *Magnetic Oscillations in Metals* (Cambridge Univ. Press, Cambridge, 1984).

[6] Lifshitz, I. M. & Kosevich, A. M. Theory of magnetic susceptibility in metals at low temperatures. *Sov. Phys. JETP* **2**, 636–645 (1956).

[7] Wang, J *et al.* Vanishing quantum oscillations in Dirac semimetal $ZrTe_5$. *Proceedings of the National Academy of Sciences* **115**, 9145–9150 (2018).

[8] Liu, Y. *et al.* Zeeman splitting and dynamical mass generation in Dirac semimetal $ZrTe_5$. *Nature Communications* **7**, 12516 (2016).

[9] Huang, X. *et al.* Observation of the Chiral-anomaly-induced negative magnetoresistance in 3D Weyl semimetal TaAs. *Phys. Rev. X* **5**, 031023 (2015).

[10] Lu, H.-Z. & Shen, S.-Q. Weak antilocalization and localization in disordered and interacting Weyl semimetals. *Phys. Rev. B* **92**, 035203 (2015).

[11] Chen, W., Lu, H.-Z. & Zilberberg, O. Weak Localization and Antilocalization in Nodal-Line Semimetals: Dimensionality and Topological Effects. *Phys. Rev. Lett.* **122**, 196603 (2019).

[12] Xiong, J. *et al.* Evidence for the chiral anomaly in the Dirac semimetal Na_3Bi. *Science* **350**, 413–416 (2015).

[13] Naumann, M. *et al.* Orbital effect and weak localization in the longitudinal magnetoresistance of weyl semimetals NbP, NbAs, TaP, and TaAs. *Phys. Rev. Materials* **4**, 034201 (2020).

[14] Nakamura, H. *et al.* Robust weak antilocalization due to spin-orbital entanglement in Dirac material Sr_3SnO. *Nature Communications* **11**, 1161 (2020).

[15] Okamoto, Y. *et al.* High-mobility carriers induced by chemical doping in the candidate nodal-line semimetal CaAgP. *Phys. Rev. B* **102**, 115101 (2020).

[16] Lee, P. A. & Ramakrishnan, T. V. Disordered electronic systems. *Rev. Mod. Phys.* **57**, 287–3317 (1985).

[17] Hikami, S., Larkin, A. I. & Nagaoka, Y. Spin orbit interaction and magnetoresistance in the two dimensional random system. *Prog. Theor. Phys.* **63**, 707–710 (1980).

[18] Lin, J. J. & Bird, J. P. Recent experimental studies of electron dephasing in metal and semiconductor mesoscopic structures. *J. Phys. Condens. Matter* **14**, R501 (2002).

[19] Xu, X. *et al.* Quantum oscillations in the noncentrosymmetric superconductor and topological nodal-line semimetal $PbTaSe_2$. *Phys. Rev. B* **99**, 104516 (2019).

[20] Pezzini, S. *et al.* Unconventional mass enhancement around the Dirac nodal loop in ZrSiS.

Nature Physics **14**, 178–183 (2018).

[21] Ali, M. N. *et al.* Butterfly magnetoresistance, quasi-2D Dirac Fermi surface and topological phase transition in ZrSiS. *Science Advances* **2** (2016).

[22] Singha, R., Pariari, A. K., Satpati, B. & Mandal, P. Large nonsaturating magnetoresistance and signature of nondegenerate Dirac nodes in ZrSiS. *Proceedings of the National Academy of Sciences* **114**, 2468–2473 (2017).

[23] Wang, X. *et al.* Evidence of Both Surface and Bulk Dirac Bands and Anisotropic Nonsaturating Magnetoresistance in ZrSiS. *Advanced Electronic Materials* **2**, 1600228 (2016).

[24] Hu, J. *et al.* Nearly massless Dirac fermions and strong Zeeman splitting in the nodal-line semimetal ZrSiS probed by de Haas–van Alphen quantum oscillations. *Phys. Rev. B* **96**, 045127 (2017).

[25] Hu, J. *et al.* Evidence of Topological Nodal-Line Fermions in ZrSiSe and ZrSiTe. *Phys. Rev. Lett.* **117**, 016602 (2016).

[26] Kumar, N. *et al.* Unusual magnetotransport from Si-square nets in topological semimetal HfSiS. *Phys. Rev. B* **95**, 121109 (2017).

[27] Okamoto, Y., Inohara, T., Yamakage, A., Yamakawa, Y. & Takenaka, K. Low carrier density metal realized in candidate line-node Dirac semimetals CaAgP and CaAgAs. *Journal of the Physical Society of Japan* **85** (2016).

[28] Neupane, M. *et al.* Observation of topological nodal fermion semimetal phase in ZrSiS. *Phys. Rev. B* **93**, 201104 (2016).

[29] Schoop, L. M. *et al.* Dirac cone protected by non-symmorphic symmetry and three-dimensional Dirac line node in ZrSiS. *Nature Communications* **7**, 11696 (2016).

Supplementary Table 1. **Characteristics of $SrAs_3$ crystals**. Hole (n_h) and electron (n_e) carrier densities and the corresponding mobilities μ_h and μ_e are listed for eleven crystals denoted as S1–S2 and A1–A9. The SdH frequency (F) taken at 2 K for $H \parallel k_y$ and ε_{F}, the energy difference between Fermi level E_{F} and the band crossing point for the corresponding poloidal orbit, are also listed.

	n_h	n_e	μ_h	μ_e	F	ε_{F}
	(10^{16}cm^{-3})	(10^{16}cm^{-3})	(10^4cm^2/V·s)	(10^4cm^2/V·s)	(T)	(meV)
S1	67.9(8)		0.38(5)		12.6(9)	-52(2)
S2	52.2(1)	0.30(4)	0.17(2)	3.5(1)	10.4(8)	-49(1)
A1	70.2(4)		0.141(8)		12.5(7)	-52(1)
A2	73.3(3)		0.090(5)		11.7(7)	-50(2)
A3	69.6(2)		0.109(5)		10.3(9)	-48(2)
A4	57.0(1)	0.11(2)	0.130(4)	4.7(2)	8.7(6)	-46(1)
A5	41.9(1)	0.56(8)	0.22(2)	3.5(1)	8.4(7)	-45(1)
A6	37.6(1)	1.1(1)	0.24(2)	2.99(8)	7.7(7)	-44(1)
A7	30.1(1)	1.6(2)	0.36(3)	3.63(2)	7.5(9)	-43(2)
A8	26.2(2)	3.0(2)	0.38(1)	5.29(4)	7.0(6)	-42(1)
A9	29.9(2)	3.3(3)	0.37(1)	6.05(4)	6.9(4)	-42(1)

Supplementary Table 2. **Quantum oscillation frequency and cyclotron masses for different orbits of the torus-shaped Fermi surface.** The quantum oscillation frequencies (F) and cyclotron masses (m^*) for the poloidal orbit α and two toroidal orbits β and δ orbits, estimated from SdH oscillations and the model Hamiltonian shown in the main text. The parameters for the model Hamiltonian are a_0 = 0.05093 eV, a_1 = -0.0046 eV·nm^2, a_2 = -0.0370 eV·nm^2, a_3 = 0.92593 eV·nm^2, b_3 = 0.6667 eV·nm, m_0 = -0.0694 eV, m_1 = 0.1019 eV·nm^2, m_2 = 0.2963 eV·nm^2, m_3 = 6.4815 eV·nm^2.

	α				β		δ	
	$H \parallel k_x$		$H \parallel k_y$		$H \parallel k_z$		$H \parallel k_z$	
	F(T)	m^*/m_e	F(T)	m^*/m_e	F(T)	m^*/m_e	F(T)	m^*/m_e
S1	7.1(7)	0.056(2)	12.6(9)	0.076(5)	32.4(8)	0.23(1)	129(1)	0.079(3)
S2	5.3(6)	0.033(1)	10.4(6)	0.080(4)	25.3(8)	0.176(8)	118(1)	
Model	4.97	0.04381	9.88	0.07437	38.15	0.246	209.5	0.181

Supplementary Table 3. **Characteristics of nodal-line semimetal candidates.** The type of nodal-line structure, quantum oscillation frequencies (F) for various magnetic field (H) directions, The low-temperature WAL contribution in the conductivity ($\Delta\sigma_{\mathrm{WAL}}/\sigma_0$), and the hole and electron carrier densities (n_h and n_e) are listed for various NLSM candidates.

Material	Nodal line type	Field direction	F (T)	$\Delta\sigma_{\mathrm{WAL}}/\sigma_0$	n_h or n_e (cm^{-3})	Reference
$PbTaSe_2$	Rings	$H \parallel c$	5.8, 80.3, 672–685, 904–910, 1249–1275		5×10^{21}	Ref. 19
ZrSiS	Cage-like	$H \parallel c$	240, 600			Ref. 20
		$H \parallel c$	23, 243			Ref. 21
		$H \parallel c$	14, 238		$6 \times 10^{19} (n_h)$<br>$1.6 \times 10^{17} (n_e)$	Ref. 22
		$H \parallel c$	18.9, 246.3			Ref. 23
		$H \parallel c$	8.4, 240		$3.59 \times 10^{20} (n_h)$<br>$3.64 \times 10^{20} (n_e)$	Ref. 24
		$H \parallel ab$	17.6, 24.5, 167.5, 170.6, 180.7			Ref. 24
ZrSiSe	Cage-like	$H \parallel c$	210			Ref. 25
		$H \parallel ab$	19.2, 22.9, 24, 126.9, 132.7, 142			Ref. 25
ZrSiTe	Cage-like	$H \parallel c$	102, 154			Ref. 25
HfSiS	Cage-like	$H \parallel c$	31, 264			Ref. 26
		$H \parallel ab$	13.5, 138.5			Ref. 26
CaAgAs	Single ring	$H \parallel c$	260		7.5×10^{19}	Ref. 4, 27
		$H \parallel ab$	50, 260			Ref. 4, 27
		$H \parallel ab$	95.0, 227.8			Ref. 3
CaAgP	Single ring			13.7%	4×10^{19}	Ref. 15
Pd-doped CaAgP	Single ring			43.6%	$2 \times 10^{20} (n_h)$<br>$2.3 \times 10^{16} (n_e)$	Ref. 15
$SrAs_3$	Single ring	$H \parallel c$	6.9–12.60	24.8–781%	$2.99\text{–}7.33 \times 10^{17} (n_h)$<br>$1.1\text{–}32.5 \times 10^{15} (n_e)$	This work

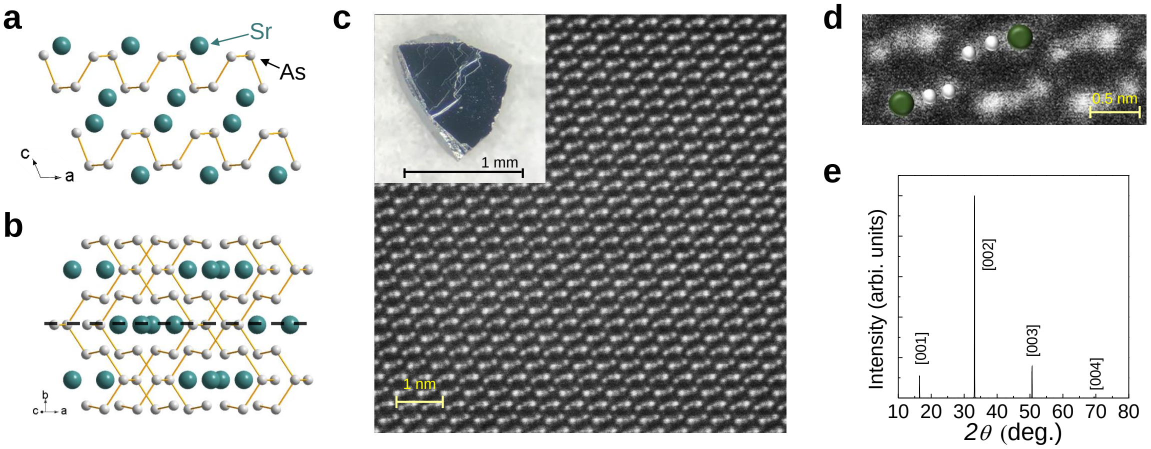

Supplementary Figure 1. **Crystal structure of $SrAs_3$ single crystals. a, b,** Crystal structure of $SrAs_3$. The side and top views along the directions normal to the a and c axes (**a**) and to the a and b axes (**b**), respectively. The dashed line in **b** indicates the mirror plane. **c, d,** The scanning transmission electron microscopy (STEM) image of $SrAs_3$ crystal in the a–c plane. Inset: optical image of a typical $SrAs_3$ single crystal. The green and white spheres in the magnified STEM image (**d**) represent Sr and As atoms, respectively. **e,** The X-ray diffraction patterns of $SrAs_3$ crystal showing $(00l)$ Bragg peaks.

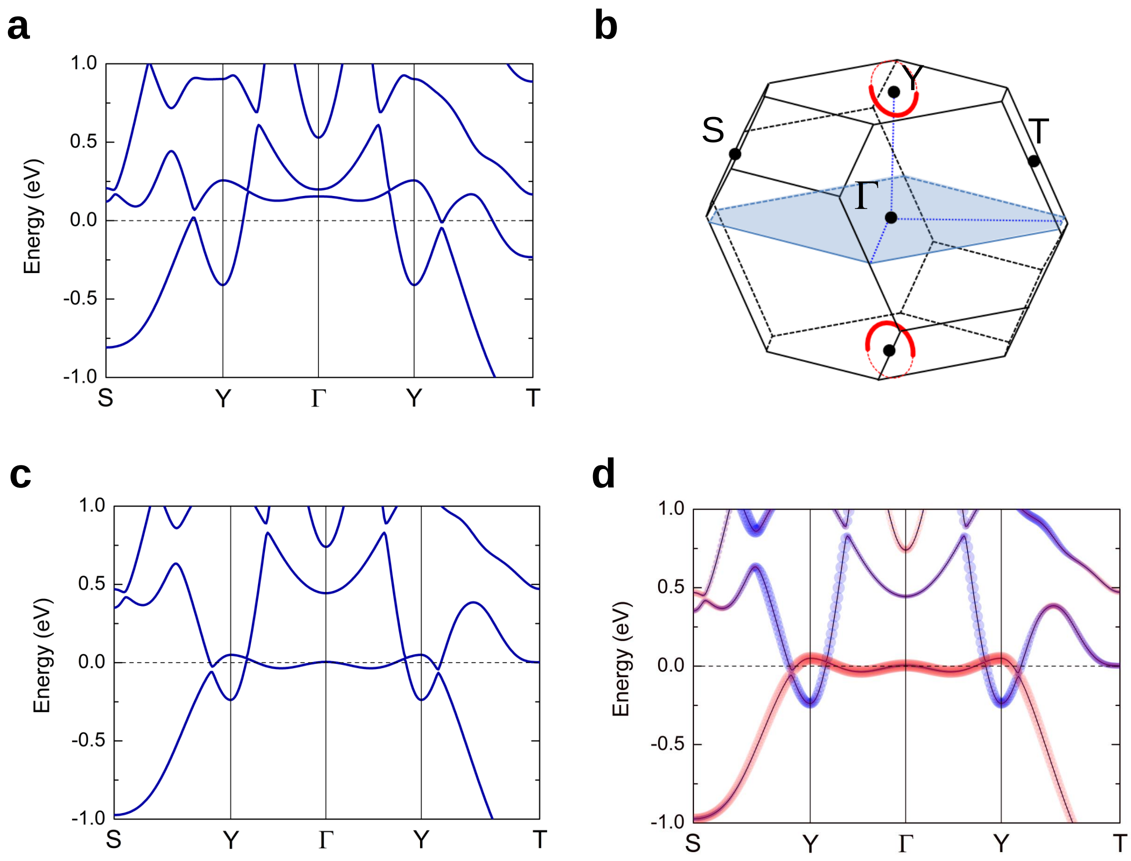

Supplementary Figure 2. **Electronic structures of $\mathbf{SrAs_3}$. a, c, d,** Electronic structure of $SrAs_3$ calculated using GGA (**a**) and mBJ functionals (**c, d**). The fat bands with blue and red represents the band from the As1 p state and the As2 p states, respectively. **b,** The Brillouin zone of $SrAs_3$ with high symmetry points. The nodal ring (red circles) is located near the Y point.

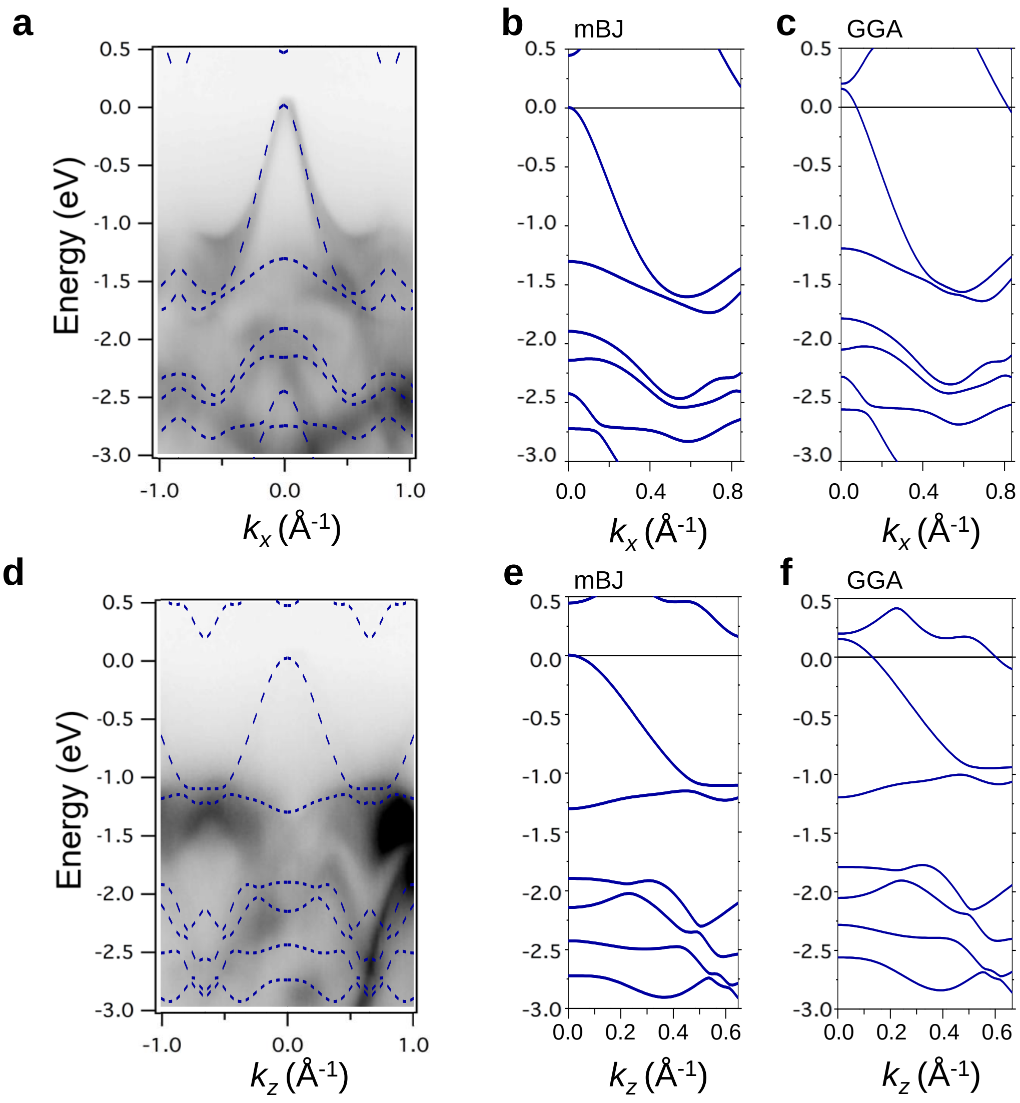

Supplementary Figure 3. **Angle resolved photoemission specroscopy and electronic structures of $SrAs_3$. a, d,** ARPES spectra taken along k_x axis (**a**) and k_z axis (**d**), together with the calculated bands using mBJ functionals (blue dashed lines). **b, c, e, f,** Band dispersion along two axes, k_x axis (**b, c**) and k_z axis (**e, f**), from mBJ calculations (**b, e**) and GGA calculations (**c, f**).

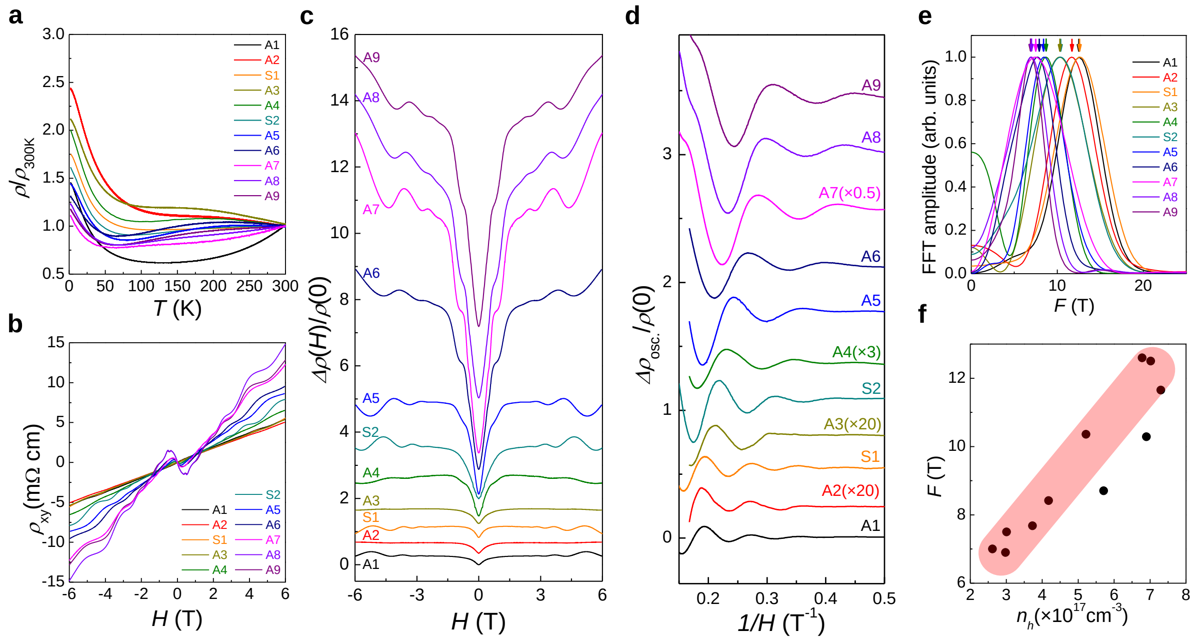

Supplementary Figure 4. **Sample dependent transport properties of $SrAs_3$ crystals. a,** The temperature dependence of the normalized in-plane resistivity (ρ) of eleven $SrAs_3$ crystals (S1-S2, and A1-A9). **b,** The magnetic field-dependent Hall resistivity (ρ_{xy}) taken at 2 K for $SrAs_3$ crystals. **c, d,** Magnetoresistivity (MR) $\Delta\rho(H)/\rho(0)$ (**c**) and SdH oscillations $\Delta\rho_{\mathrm{osc.}}(H)/\rho(0)$ (**d**) of $SrAs_3$, taken at 2 K for $H \parallel k_y$. **e,** The normalized fast Fourier transform (FFT) amplitudes of Shubnikov-de Haas (SdH) oscillations in **d**. **f,** SdH frequency (F) and the hole carrier density (n_h) for various $SrAs_3$ crystals.

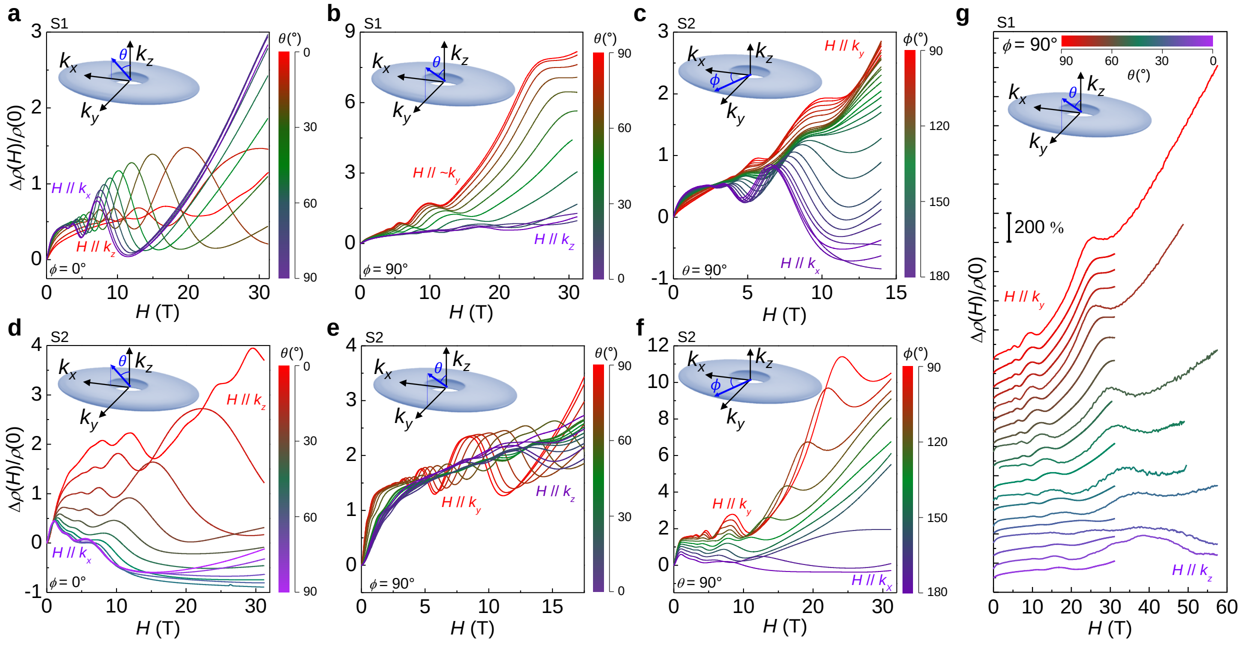

Supplementary Figure 5. **The angle-dependent SdH oscillations of $SrAs_3$ crystals.** The angle-dependence magnetoresistivity ($\Delta\rho(H)/\rho(0)$) of different samples, S1 (**a, b, c, g**) and S2 (**d, e, f**), taken at around 2 K with different field orientations in the (k_x,k_z) plane (**a, d**), (k_y,k_z) plane (**b, e, g**), and (k_x,k_y) plane (**c, f**). Magnetoresistivity was taken with static magnetic fields up to 31.6 T and pulsed magnetic fields up to 57.6 T in **g**.

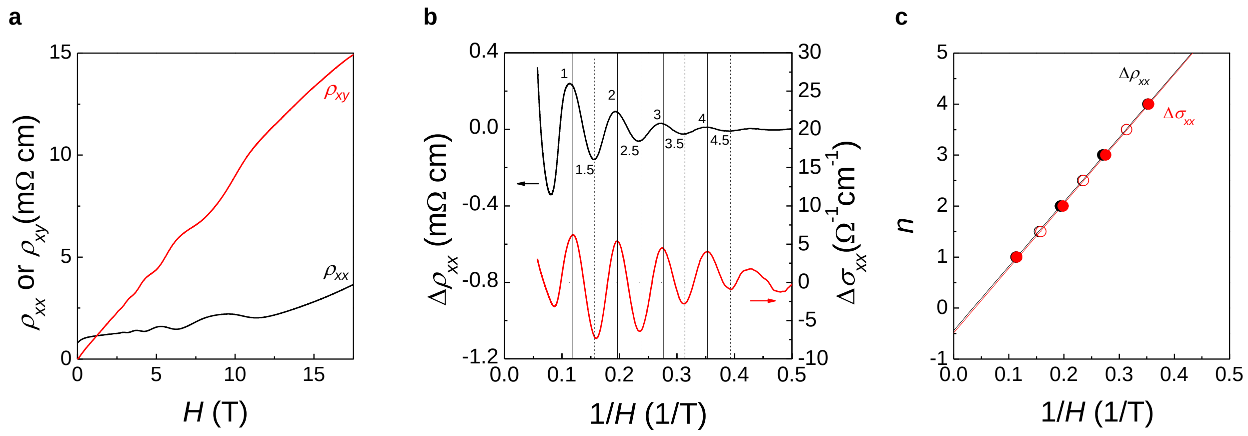

Supplementary Figure 6. **Comparison of SdH oscillations in ρ_{xx} and σ_{xx}. a,** The magnetic field-dependent longitudinal resistivity (ρ_{xx}) and Hall resistivity (ρ_{xy}) at $H \parallel k_y$ and 1.4 K for S1. **b,** Shubnikov-de Haas (SdH) oscillations in magnetic field-dependent resistivity ($\Delta\rho_{xx}$) and conductivity ($\Delta\sigma_{xx}$). The maxima and minima from $\Delta\rho_{xx}$ and $\Delta\sigma_{xx}$ coincide with each other, which are assigned with integer and half-integer of the Landau index, indicated by the vertical lines. **c,** Landau fan diagram with maxima (solid circles) and minima (open circles) of $\Delta\rho_{xx}$ and $\Delta\sigma_{xx}$. The linear fits to the data from $\Delta\rho_{xx}$ and $\Delta\sigma_{xx}$ yield the same intercept on the Landau index axis.

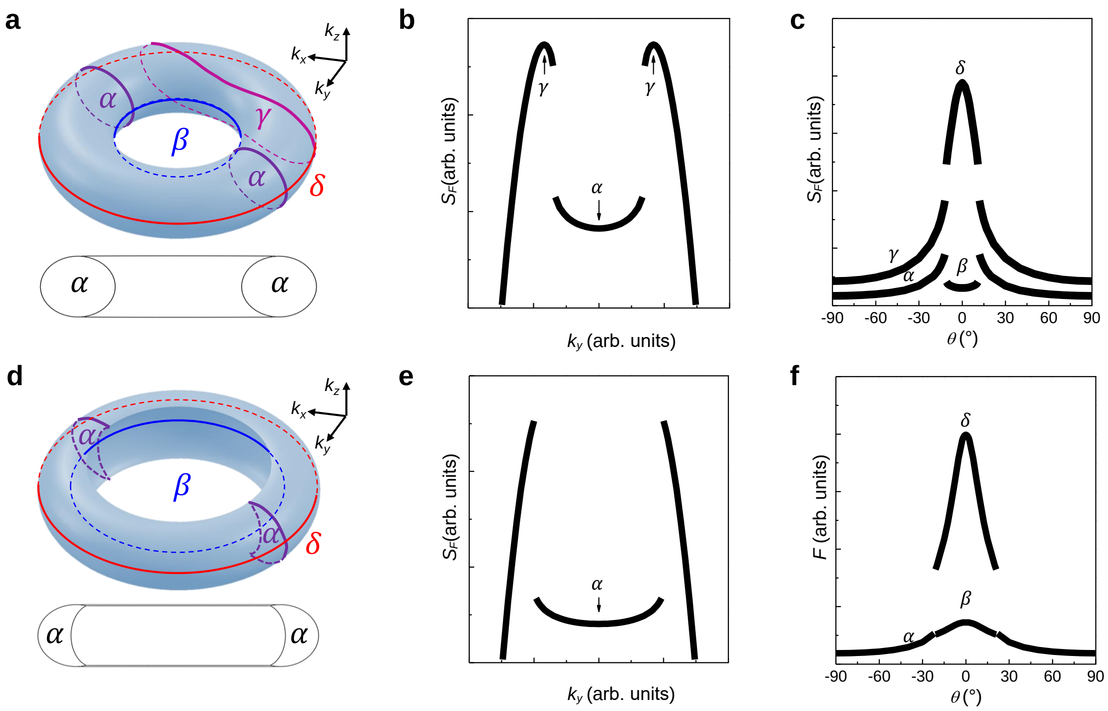

Supplementary Figure 7. **Comparison of two different types of torus-shaped Fermi surfaces. a, d,** The torus-shaped Fermi surfaces with an elliptical-shaped (**a**) and a crescent-shaped (**d**) poloidal cross-sections. Two extremal orbits, generated by intersecting planes normal to the k_y axis and parallel to the k_z axis (spiric section), correspond to the α orbit at $k_y = 0$ and the γ orbit at $k_y \neq 0$. Two inner and outer toroic orbits in the (k_x,k_y) plane correspond to the β and δ orbits, respectively. Note that the γ orbit is missing for the FS of **d**. The corresponding poloidal cross-sections are also shown. **b, e,** The area of the cross-section (S_F), generated by spiric section at different k_y, for the FSs shown in **a** and **d**. The extremal positions for the α and γ orbits are indicated by the arrows (**b**) for the FS shown in **a**. The extremal position is located only at $k_y =$ 0 (**e**) for the FS of **d**. **c, f,** SdH frequencies (F) as a function of the polar angle (θ) with respect to the k_z axis for the FS of **a** and **d**. At every angles, two SdH frequencies, either α and γ, or β and δ, are present (**c**) for the FS of **a**. For the FS of **d**, the β and δ frequencies show the similar angle dependence, in the absence of the γ frequency (**f**).

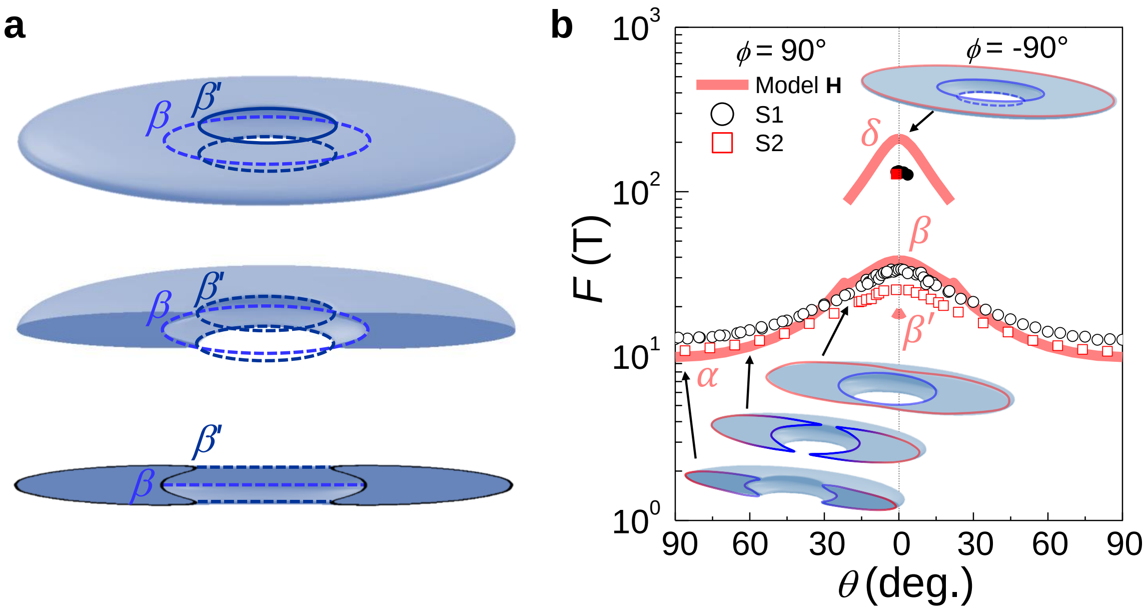

Supplementary Figure 8. **Splitting of the inner toroidal orbits in $SrAs_3$.** **a,** Two inner toroidal orbits, denoted as β and β', in the torus Fermi surface with crescent-shaped cross-section, seen from different view angles. **b,** The expected SdH frequency (F) of the β' orbit from the model Hamiltonian in the main text.

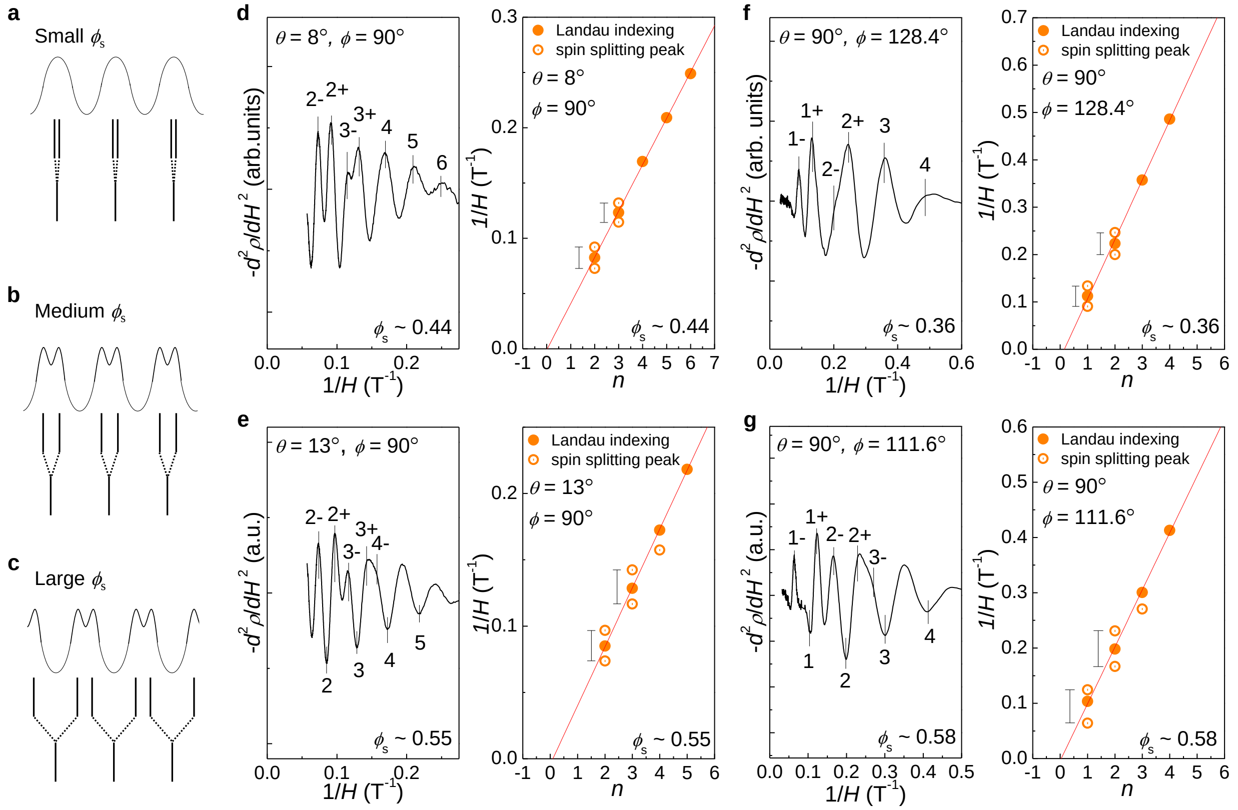

Supplementary Figure 9. **Landau fan diagram with Zeeman splitting in SrAs$_3$. a–c,** The schematic illustrations of quantum oscillation peaks depending of the size of the spin splitting phase ϕ_s. The vertical bars under oscillation oscillation curves correspond to the Zeeman-split levels from the spin-degenerate Landau level. **d–g,** The quantum oscillations in the second derivative of resistivity, $-d^2\rho/dH^2$, and corresponding Landau fan diagram for S2. The vertical bars in the Landau fan diagram show the spin splitting spacing, which is expected to be field-independent. The magnetic field directions are on the (k_y, k_z) plane (**d, e**) or (k_x, k_y) plane (**f, g**).

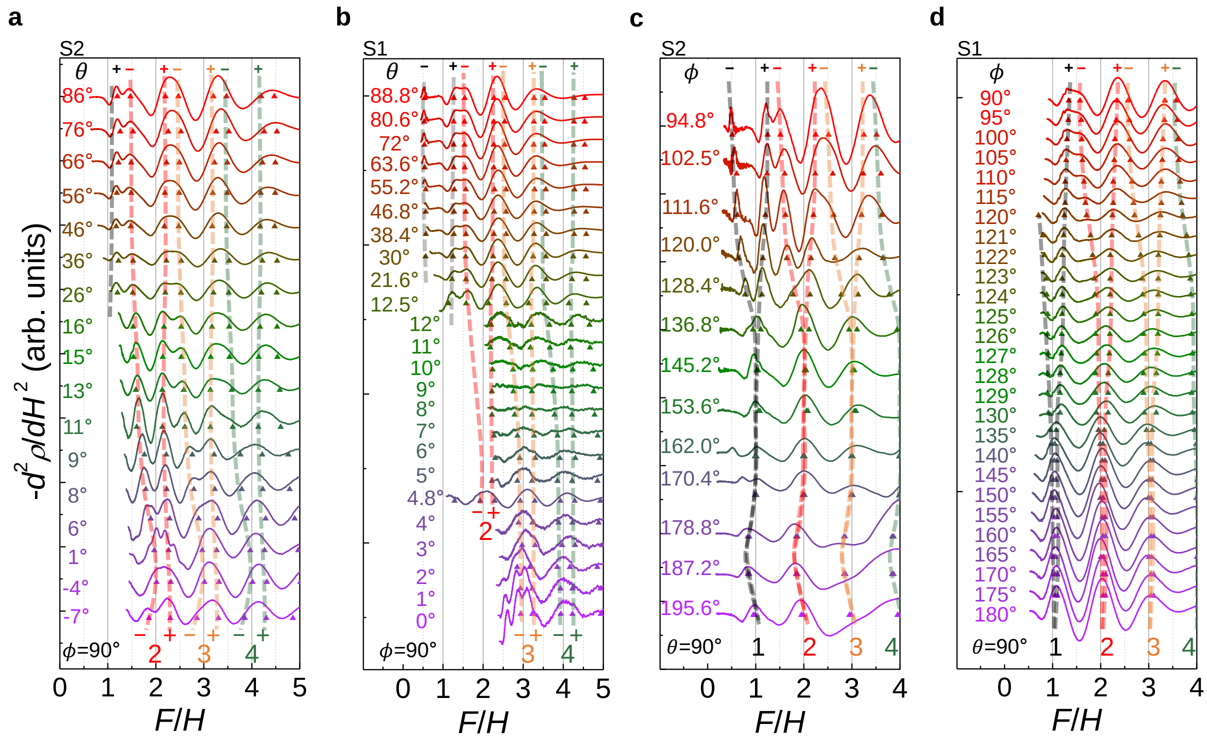

Supplementary Figure 10. **Shubnikov-de Hass oscillations of $SrAs_3$ near the quantum limit. a–d,** The second derivative of $\rho(H)$, $-d^2\rho/dH^2$, as a function of the nomalized F/H for various magnetic field orientations with different polar (θ) angles (**a, b**) and azimuthal (ϕ) angles (**c, d**) for S2 (**a, c**) and S1 (**b, d**). The spin splitting peaks of SdH oscillations are indicated by triangle symbols. The shaded dashed lines correspond to the spin-split Landau levels, indicated by the color-coded integer index and the + and − symbols.

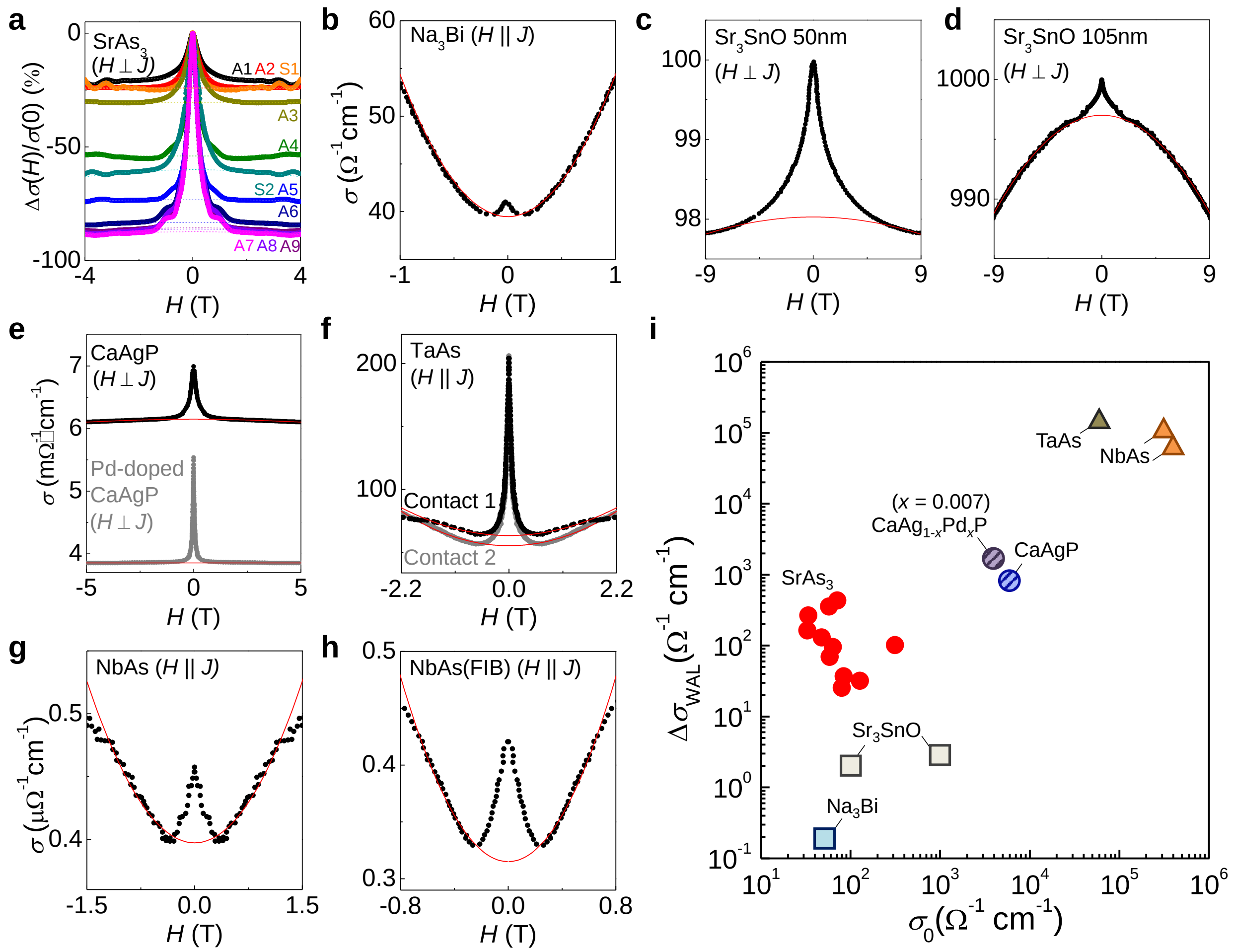

Supplementary Figure 11. **Weak antilocalization analysis of various topological semimetals. a,** The magnetic field dependent conductivity ratio $\Delta\sigma(H)/\sigma(0)$ in $SrAs_3$. **b–h,** The magnetic field dependent conductivity $\sigma(H)$ in various topological semimetals at low-temperatures [12–15]. T fit to the high field data is presented with the dashed line (**a**) or the red solid line (**b**–**h**). **i,** The excess conductivity $\Delta\sigma_{\mathrm{WAL}}$ as a function of σ_0 for various topological semimetals.

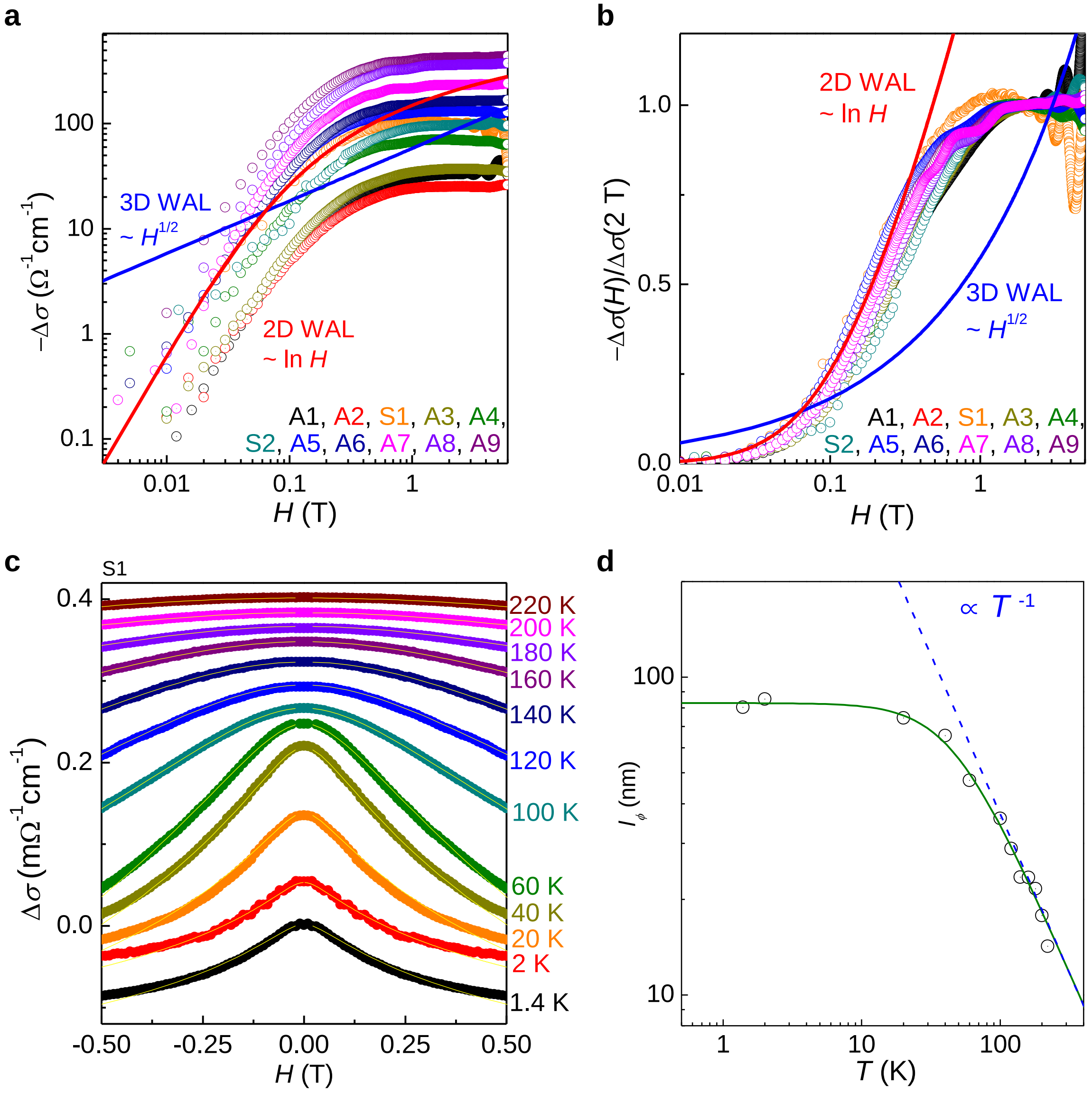

Supplementary Figure 12. **The dimensionality of weak antilocalizatoin in $SrAs_3$ a, b,** The log-log plot of magnetoconductivity $-\Delta\sigma(H)$ (**a**) and log plot of normalized magnetoconductivity (**b**) for eleven $SrAs_3$ crystals with 2D WAL (red line) and 3D WAL (blue line) fittings to S1. **c** Temperature dependent magnetoconductivity of S1 with HLN equation fitting (yellow line). **d** Temperature-dependent phase coherence length l_ϕ for S1, following T^{-1} dependence (blue dashed line) at high temperatures. The fit to the 2D WAL model is also shown (green solid line).
